# Gas-Phase Biosensors (Bio-Sniffers) for Measurement of 2-Nonenal, the Causative Volatile Molecule of Human Aging-Related Body Odor

**DOI:** 10.3390/s23135857

**Published:** 2023-06-24

**Authors:** Kenta Iitani, Hidehisa Mori, Kenta Ichikawa, Koji Toma, Takahiro Arakawa, Yasuhiko Iwasaki, Kohji Mitsubayashi

**Affiliations:** 1 Department of Biomedical Devices and Instrumentation, Institute of Biomaterials and Bioengineering, Tokyo Medical and Dental University (TMDU), Tokyo 101-0062, Japan; i.bdi@tmd.ac.jp (K.I.); ichikawa.kenta@tmd.ac.jp (K.I.); k-toma@shibaura-it.ac.jp (K.T.); arakawath@stf.teu.ac.jp (T.A.); 2 Graduate School of Medical and Dental Sciences, Tokyo Medical and Dental University (TMDU), Tokyo 113-8510, Japan; morihidehisa@gmail.com; 3 Department of Electronic Engineering, College of Engineering, Shibaura Institute of Technology, Tokyo 135-8548, Japan; 4 Department of Electric and Electronic Engineering, Tokyo University of Technology, Tokyo 192-0982, Japan; 5 Faculty of Chemistry, Materials and Bioengineering, Kansai University, Osaka 564-8680, Japan; yasu.bmt@kansai-u.ac.jp

**Keywords:** gas-phase biosensor, *trans*-2-nonenal, body odor, aging, fluorescence

## Abstract

The molecule 2-nonenal is renowned as the origin of unpleasant human aging-related body odor that can potentially indicate age-related metabolic changes. Most 2-nonenal measurements rely on chromatographic analytical systems, which pose challenges in terms of daily usage and the ability to track changes in concentration over time. In this study, we have developed liquid- and gas-phase biosensors (bio-sniffers) with the aim of enabling facile and continuous measurement of *trans*-2-nonenal vapor. Initially, we compared two types of nicotinamide adenine dinucleotide (phosphate) [NAD(P)]-dependent enzymes that have the catalytic ability of *trans*-2-nonenal: aldehyde dehydrogenase (ALDH) and enone reductase 1 (ER1). The developed sensor quantified the *trans*-2-nonanal concentration by measuring fluorescence (excitation: 340 nm, emission: 490 nm) emitted from NAD(P)H that was generated or consumed by ALDH or ER1. The ALDH biosensor reacted to a variety of aldehydes including *trans*-2-nonenal, whereas the ER1 biosensor showed high selectivity. In contrast, the ALDH bio-sniffer showed quantitative characteristics for *trans*-2-nonenal vapor at a concentration range of 0.4–7.5 ppm (with a theoretical limit of detection (LOD) and limit of quantification (LOQ) of 0.23 and 0.26 ppm, respectively), including a reported concentration (0.85–4.35 ppm), whereas the ER1 bio-sniffer detected only 0.4 and 0.8 ppm. Based on these findings, headspace gas of skin-wiped alcohol-absorbed cotton collected from study participants in their 20s and 50s was measured by the ALDH bio-sniffer. Consequently, age-related differences in signals were observed, suggesting the potential for measuring *trans*-2-nonenal vapor.

## 1. Introduction

Humans release a diverse array of volatile organic compounds (VOCs) [1]. Since some of those human-borne VOCs (H-VOCs) include metabolites produced in human biological activities, studies of them have been actively conducted to realize disease screening and metabolic monitoring [2,3,4]. While the majority of the H-VOCs are devoid of odor, there are specific odorous chemicals within H-VOCs that contribute to body odor [5,6,7]. Among those components are those associated with aging [8,9]; 2-nonenal, with an odor described as greasy and grassy, is typical. This odorous molecule is known as one of the causatives of unpleasant body odor that is emitted by people over around 40 years old, and its concentration is reported to increase with aging [10]. In brief, the generation of 2-nonenal is influenced by age-related metabolic changes through the following mechanisms. Initially, the formation of lipid peroxides occurs when unsaturated fatty acids react with reactive oxygen species (ROS) present in skin surface lipids [11]. As individuals age, the quantity of ROS increases due to alterations in intracellular metabolism and mitochondrial function [12]; simultaneously, the concentration of antioxidant enzymes and molecules in the body decreases [13]. This combination makes it difficult to suppress the formation of lipid peroxide by ROS. Consequently, these lipid peroxides cause oxidative degradation of ω7-monounsaturated fatty acids such as palmitoleic acid and vaccenic acid in skin surface lipids, ultimately leading to the production of 2-nonenal. Additionally, along with the lipid peroxides, the levels of ω7-monounsaturated fatty acids also increase in skin surface lipids as individuals age [14]. Therefore, 2-nonenal serves as a discernible odorous marker of the aging process. Although the precise mechanism underlying the age-related increase in ω7-monounsaturated fatty acids remains unclear, another study [15] supports the findings of Haze et al. [10] by reporting similar ager-related increases in these fatty acids.

As previously mentioned, 2-nonenal possesses a potent odor, and individuals often utilize deodorants to maintain positive social interactions [16,17,18]. However, self-assessment of one’s own body odor can be challenging due to olfactory adaptation [19]. In addition to the interpersonal aspect of body odor, there is the potential for evaluating changes in the metabolic capacity associated with aging based on the concentration of 2-nonenal. Therefore, methods to quantitatively measure its concentration are required. Most of the methods use gas chromatography–mass spectrometry and high-performance liquid chromatography. While numerous studies have focused on the measurement of 2-nonenal in food products [20,21,22,23,24,25], there has been relatively less research conducted specifically on human-borne 2-nonenal [26,27]. Given that laboratory-grade analytical instruments are unsuitable for daily monitoring of body odor and metabolic status due to their size, cost, and complexity, there is a demand for small and facile operative 2-nonenal gas sensors. Sensor devices resembling electronic noses, comprising multiple gas sensors [28,29,30,31,32] as well as plasmonic sensors [33], have been developed and commercialized for evaluation of body odor. However, to the best of our knowledge, there is currently no continuous gas sensor available that can selectively and quantitatively measure 2-nonenal.

We have developed a fiber-optic biochemical gas sensor (bio-sniffer) using nicotinamide adenine dinucleotide (NAD)-dependent dehydrogenase. In this particular study, our initial investigation involved comparing different enzymes capable of catalyzing *trans*-2-nonenal in the liquid phase. Subsequently, a bio-sniffer for *trans*-2-nonenal was developed, and the basic characteristics were evaluated. Furthermore, we employed the ALDH bio-sniffer to measure the headspace gas emitted from cotton wipes used on various body areas of study participants in their 20s and 50s.

## 2. Materials and Methods

### 2.1. Materials and Reagents

Aldehyde dehydrogenase (ALDH, cat# 10171832001, from *yeast*, EC 1.2.1.5, with a specific activity of 20 units/mg protein, Roche, Switzerland) and enone reductase 1 (ER1, Lot# AE-001, with a specific activity of 1.3 units/mg powder, Daicel, Tokyo, Japan) [34] were utilized without additional purification. The oxidized form of nicotinamide adenine dinucleotide (NAD^+^) and reduced form of nicotinamide adenine dinucleotide phosphate (NADPH) were purchased from Oriental Yeast, Japan. A copolymer consisting of 2-methacryloyloxy ethyl phosphorylcholine (MPC) and 2-Ethylhexyl methacrylate (EHMA), known as PMEH [35], was synthesized within the laboratory and employed for enzyme immobilization [36]. Disodium hydrogen phosphate and potassium dihydrogen phosphate were used to prepare potassium buffer (PB). The pH of buffer was adjusted by a pH meter (D-25, Horiba, Kyoto, Japan). For sensor characterization, *trans*-2-nonenal (#255653-5G, Sigma-Aldrich, Burlington, MA, USA), acetaldehyde (#015-09576, FUJIFILM Wako, Osaka, Japan), hexanal (#085-02633, FUJIFILM Wako, Osaka, Japan), heptanal (#084-00143, FUJIFILM Wako, Osaka, Japan), octanal (#150-00053, FUJIFILM Wako, Osaka, Japan), nonanal (#149-06002, FUJIFILM Wako, Osaka, Japan), decanal (#045-21532, FUJIFILM Wako, Osaka, Japan), ethyl vinyl ketone (#L06829.06, Thermofisher, Waltham, MA, USA), ethanol (#14033-00, Kanto Chemical, Tokyo, Japan), and acetone (#016-00346, FUJIFILM Wako, Osaka, Japan) were employed.

### 2.2. trans-2-Nonenal Biosensor Using NAD(P)H-Dependent Enzymes

**Figure 1:**
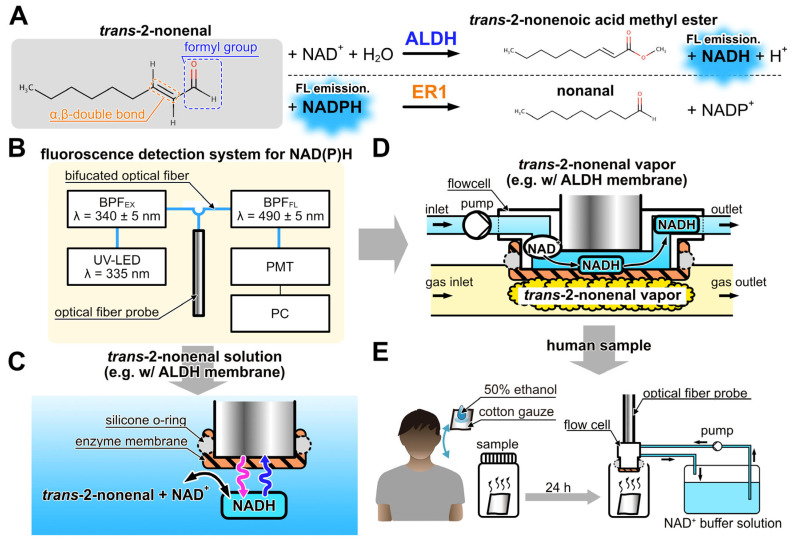
(**A**) Detection principle of *trans*-2-nonenal based on the catalytic reaction of ALDH and ER1. (**B**) Schematic diagram of the fiber-optic fluorescence measurement system of NAD(P)H. (**C**) Detection scheme of *trans*-2-nonenal in liquid phase with ALDH-immobilized membrane combined with the fiber-optic system. (**D**) Detection scheme of *trans*-2-nonenal vapor with the flow cell. (**E**) Measurement scheme of the human sample with bio-sniffer.

Figure 1A illustrates the enzymatic reactions of ALDH and ER1 with *trans*-2-nonenal as a substrate, based on the literature [37,38]. Both enzymatic reactions used NAD(P) as an electron carrier. In the reactions, NAD(P) can exist in the oxidized form NAD(P)^+^ and reduced form NAD(P)H. NAD(P)H absorbs UV light at 340 nm and emits fluorescence at 490 nm. The fluorescence intensity of NAD(P)H varies with its concentration, allowing enzymatic reactions to be monitored by measuring the fluorescence intensity of NAD(P)H. In the ALDH-catalyzed reaction, NADH was produced through the oxidation of *trans*-2-nonenal, which takes place by recognizing the formyl group. On the other hand, in the ER1-catalyzed reaction, NADPH is utilized during the reduction of *trans*-2-nonenal, which proceeds by recognizing the α-β double bond. Consequently, *trans*-2-nonenal can be detected by an increase in fluorescence intensity with ALDH and a decrease in fluorescence intensity with ER1. Figure 1B illustrates the fluorescence detection system used in this study as well as the previous studies [39,40]. The system consists of UV-LED (λ = 335 nm, Sensor Electronic Technology, Newbury Park, CA, USA), an excitation band-pass filter (BPF_EX_, 340 ± 5 nm, Asahi spectra, Tokyo, Japan), an optical fiber probe (F1000-900 PROBE, Ocean Insight, Orlando, FL, USA), a fluorescence band-pass filter (BPF_FL_, 490 ± 5 nm, Asahi spectra, Tokyo, Japan), and a photomultiplier tube (PMT, C9692, Hamamatsu photonics, Tokyo, Japan). These components are connected using a bifurcated Y-shape optical fiber (BIF600-UV/VIS, Ocean Insight, Orlando, FL, USA). The PMT was connected to a PC with photon-counting mode to measure NAD(P)H fluorescence emitted at the tip of the optical fiber probe. Based on the fluorescence detection system, liquid and vapor phase biosensors for *trans*-2-nonenal were developed.

The *trans*-2-nonenal biosensors of the liquid and vapor phase used enzyme-immobilized membranes located on the tip of the optical fiber probe (Figure 1C) or the flow cell (Figure 1D), respectively. The ALDH-immobilized membrane was prepared as follows (see Figure S1). First, 2 cm × 2 cm hydrophilic polytetrafluoroethylene membrane (H-PTFE, thickness 80 μm, pore size 0.2 μm, Merck Millipore, St. Louis, MO, USA) was rinsed with ultrapure water and dried. Then, a mixture of ALDH solution and PMEH (10 *w*/*w*% in ethanol, 10 μL/cm^2^) was spread on an H-PTFE membrane by spatula, with the enzyme quantity being 2.1 units/cm^2^. Then, the prepared membrane was dried for 3 h at 4 °C. Afterward, the unentrapped ALDH was rinsed with phosphate buffer (PB). Similarly, the ER1-immobilized membrane was prepared following the same procedure as the ALDH-immobilized membrane. The quantity of ER1 used was 1.3 units/cm^2^.

### 2.3. Evaluation of ALDH or ER1 Biosensors on Trans-2-Nonenal Solution

As shown in Figure 1C, ALDH or ER1 membrane was attached to the tip of the optical fiber probe by using a silicone O-ring and then dipped in the coenzyme solution. In the evaluation of the ALDH biosensor, 10 mM NAD^+^ solution (in 300 μL of PB at pH 8.0, 100 mM) was poured into a cuvette, and *trans*-2-nonenal solution diluted with 80% of ethanol diluted by PB was added dropwise every 5 min to reach a final concentration of 1 nM–10 mM. The ER1 biosensor was evaluated in the same way as the ALDH biosensor, except that 100 μM NADPH (in 300 μL of PB at pH 7.0, 100 mM) solution was used as the coenzyme solution in the cuvette.

To assess the selectivity of the ALDH and ER1 biosensors, coenzyme solutions compatible with each enzyme were employed. *trans*-2-nonenal, along with various aldehydes (acetaldehyde, hexanal, heptanal, octanal, nonanal, and decanal), were prepared at a concentration of 10 μM in 80% ethanol solution. Additionally, ethyl vinyl ketone (which is the primary substrate of ER1), ethanol (employed as a solvent), and acetone (a typical skin gas component) were likewise evaluated at 10 μM each.

### 2.4. Measurement of trans-2-Nonenal Vapor

As depicted in Figure 1D, a gas-liquid flow cell was employed in the *trans*-2-nonenal bio-sniffer by attaching it to the optical fiber probe. The space between an enzyme-immobilized membrane and the opening of the optical fiber probe served as a liquid channel for the coenzyme solution. The flow cell was fabricated by machining an acrylic rod (O.D. 12 mm) with a 1.0 mm diameter hole on the side. The gap between the probe end and the enzyme-immobilized membrane was set at 0.4 mm. Figure 1D provides a schematic representation of the flow cell featuring the ALDH-immobilized membrane. NADH was generated on the ALDH-immobilized membrane by loading the *trans*-2-nonenal vapor, and then NADH was immediately discharged by the delivery of fresh coenzyme solution. Due to the constant flow rate of the coenzyme solution, the higher concentration of *trans*-2-nonenal vapor resulted in an accelerated NADH generation rate, thereby yielding an elevated fluorescence intensity. Conversely, in the case of the bio-sniffer with ER1 membrane, the fluorescence intensity diminished with increasing *trans*-2-nonenal concentration. The flow cell effectively maintained the moisture of the enzyme membrane, rendering it less susceptible to samples with high humidity, such as skin gases. Previous iterations of bio-sniffers demonstrated accurate quantification of molecule concentrations in exhaled breath, employing a calibration curve derived from dry standard gas [41]. This substantiates the limited influence of humidity in the bio-sniffer.

The *trans*-2-nonenal vapor was prepared by using polyvinylidene difluoride (PVDF) sampling bags. In the experiment, a pure *trans*-2-nonenal solution was injected into a PVDF bag using a micro syringe (7000.5 KH, Hamilton, Reno, NV, USA) to adjust a final gas concentration of 0.4 ppm to 10 ppm and then heated in a water bath at 60 °C for 20 min to facilitate vaporization of the *trans*-2-nonenal and obtain the standard vapor. The injection volume of *trans*-2-nonenal solution was based on the calculation outlined in Equation (1), taking into consideration the principles of the ideal gas law:

(1) $$C=\frac{\frac{D\times V_{inj}}{MW\;}\times \frac{273+T}{273}\times \frac{1013}{P}\times 22.4\times 10^{3}}{V_{dilu}}$$

where *C* is concentration (ppm), *MW* is molecular weight (g/mol), *D* is density (g/mL), *V_inj_* is injection volume (μL), *T* is temperature (°C), *P* is atmospheric pressure (hPa), and *V_dilu_* is dilution gas volume (L).

The gas measurement setup is illustrated in Figure S2. A peristaltic pump (MP-1000-H, Tokyo Rikakikai, Tokyo, Japan) was used to flow over the coenzyme solution at 1.4 mL/min. The coenzyme solution was changed depending on the enzyme as follows: 1 mM NAD^+^ solution (in PB at pH 8.0, 100 mM) for ALDH and 50 μM NADPH solution (in PB at pH 7.0, 100 mM) for ER1. The *trans*-2-nonenal vapor in the PVDF bag was delivered to the bio-sniffer at a flow rate of 140 mL/min using a diaphragm pump (DSA-3-12, Denso Sangyo, Kobe, Japan). PTFE tubing with low adsorption characteristics was used for the gas piping. The response of the bio-sniffer was evaluated by first loading dry clean air, then switching to *trans*-2-nonenal vapor, and then switching back to dry clean air to observe temporal changes in fluorescence intensity.

### 2.5. Measurement of Human Samples

The experiment was conducted with the approval of the ethics review committee at Tokyo Medical and Dental University (TMDU) in accordance with the latest version of the Declaration of Helsinki (approval number 2012-06). Prior to the experiment, participants were provided with a detailed explanation regarding the methods and significance of the study, and written informed consent was obtained from participants. The study included a total of six participants, consisting of two individuals in their 50s and four individuals in their 20s. Initially, participants were requested to complete a questionnaire regarding their gender, age, and body mass index (BMI). To collect samples, the nape, axilla, back of the ear, and shoulder of each participant were gently wiped using cotton gauze soaked in a 500 μL solution of 50% ethanol. The wiped samples were sealed in 9 mL screw bottles and placed in a dark location for one day to prepare headspace gas. The human samples and a blank sample for one participant were measured by the ALDH bio-sniffer, as shown in Figure 1E. The measurement was performed with a sample application time of 10 min, and 10 min was taken after application without sample gas.

## 3. Results and Discussion

### 3.1. Responses of ALDH and ER Biosensors

**Figure 2:**
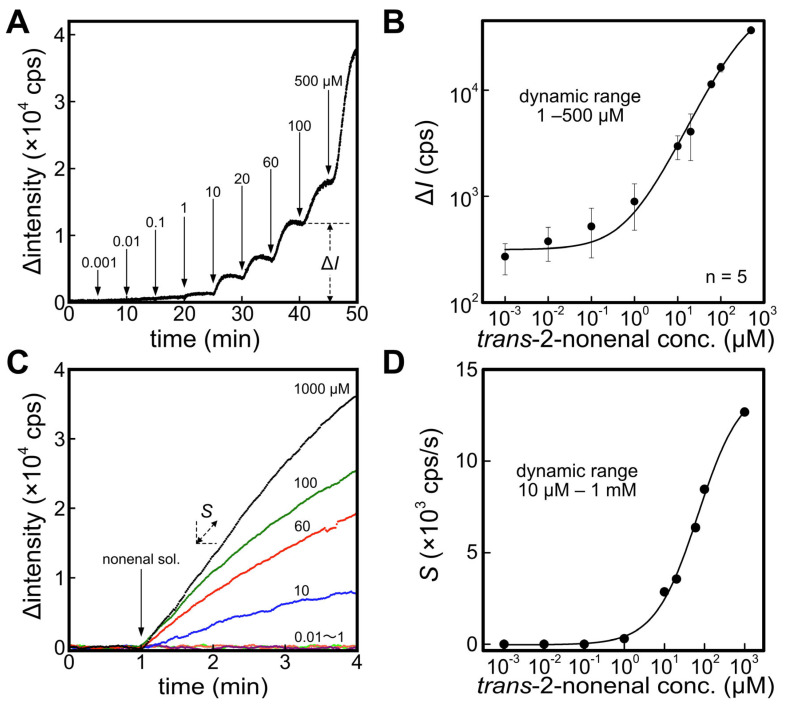
(**A**) Time course of fluorescence intensity with applying different concentrations of *trans*-2-nonenal solution against an ALDH biosensor. The difference between baseline and after applying each concentration of *trans*-2-nonenal was defined to Δ*I*. (**B**) Calibration curve of *trans*-2-nonenal solution with the ALDH biosensor. (**C**) Response curves of an ER1 biosensor against different concentrations of *trans*-2-nonenal. Slope of the fluorescence change was defined to *S*. (**D**) Calibration curve of *trans*-2-nonenal solution with the ER1 biosensor.

Figure 2A shows the response of the ALDH biosensor against the different concentrations of *trans*-2-nonenal solution. An increase in fluorescence intensity from the baseline (fluorescence intensity of 0–5 min) was observed by adding *trans*-2-nonenal solution, attributed to the catalytic reaction facilitated by ALDH. Furthermore, a convergence towards a constant fluorescence intensity corresponding to *trans*-2-nonenal concentration was observed. Subsequently, the output Δ*I* was defined as the difference between the average fluorescence intensity at 4–5 min after injection of *trans*-2-nonenal and the baseline. Figure 2B depicts a correlation between Δ*I* and *trans*-2-nonenal concentration. The dynamic range of the ALDH biosensor against *trans*-2-nonenal was 1–500 μM with the calibration curve (R^2^ = 0.998) shown in Equation (2).

(2) $$\Delta I\;\left(\mathrm{cps}\right)=\frac{315.5-65.3\times 10^{3}}{1+{\left(\left[\mathrm{trans}-2-\mathrm{nonenal}\;\mathrm{conc}.\;(\mu M)\right]/384.3\right)}^{0.85}}+65.3\times 10^{3}$$

Figure 2C displays the temporal changes in fluorescence upon the addition of *trans*-2-nonenal solution to the cuvette containing the immersed ER1 biosensor. Although the actual fluorescence intensity decreases with the introduction of *trans*-2-nonenal, the graph presents an inverted representation of the amount of change in fluorescence intensity. In comparison to the ALDH biosensor, the ER1 biosensor exhibited a lower responsiveness to *trans*-2-nonenal and did not exhibit a constant fluorescence intensity. Consequently, the slope of Δintensity *S* was employed to evaluate the quantitative characteristic. As a result, the dynamic range of the ER1 biosensor was 10 μM–1 mM, supported by the calibration curve (R^2^ = 0.996) outlined in Equation (3). A potential factor contributing to this behavior could be diminished enzyme activity resulting from the presence of 80% ethanol used as the solvent for *trans*-2-nonenal. The tolerance of enzymes to organic solvents can vary significantly depending on their type and origin. Generally, exposure to organic solvents leads to permanent conformational changes in enzymes, resulting in a loss of activity. Previous studies have demonstrated that notable alterations in protein conformation commence at ethanol concentrations above 20%, with the most pronounced effects observed at 50% ethanol [42,43,44,45]. However, this hypothesis has not been verified and is a future issue.

(3) $$S\;\left(\mathrm{cps}/s\right)=\frac{-0.03-14.2}{1+{\left(\left[\mathrm{trans}-2-\mathrm{nonenal}\;\mathrm{conc}.\;(\mu M)\right]/69.2\right)}^{0.80}}+14.2$$

### 3.2. Selectivity of ALDH and ER Biosensors

**Figure 3:**
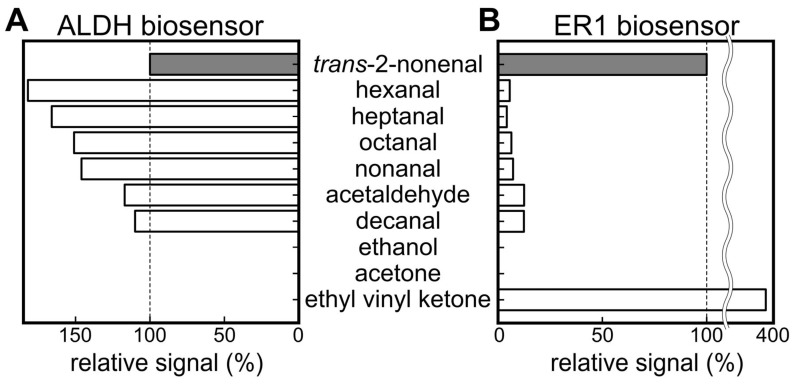
Results of selectivity test of (**A**) ALDH biosensor and (**B**) ER1 biosensor against various molecules.

Figure 3A,B presents the selectivity against *trans*-2-nonenal in comparison to various aldehydes and other molecules. The graphs display the relative output values of each biosensor, with *trans*-2-nonenal setting the reference at 100%. In the case of the ALDH biosensor, a higher fluorescence signal was observed for hexanal, heptanal, octanal, nonanal, acetaldehyde, and decanal than for *trans*-2-nonenal. It was reported that relatively high concentrations of hexanal, heptanal, octanal, nonanal, acetaldehyde, and decanal among aldehydes were contained in the VOC composition of healthy people [46]. Therefore, while the ALDH biosensor could be used to detect *trans*-2-nonenal, the measurement of human samples was likely to be influenced by other aldehydes. In contrast, the ER1 biosensor also showed output for other aldehydes, but the relative values were less than 13% for each component, which resulted in selectivity based on substrate specificity.

### 3.3. Quantitative Characteristics of ALDH Bio-Sniffer against trans-2-Nonenal

**Figure 4:**
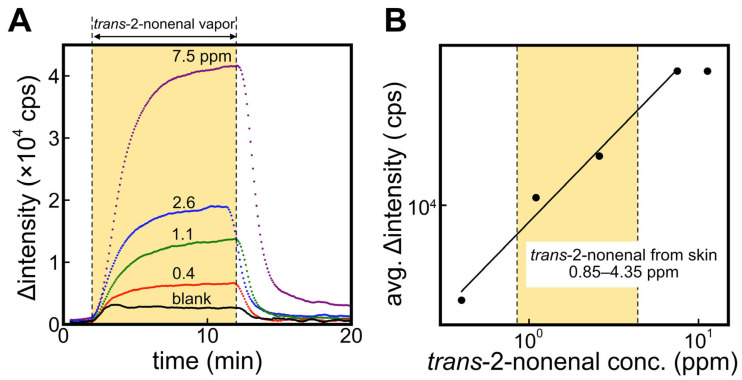
(**A**) Time course of fluorescence intensity by applying *trans*-2-nonenal vapor against ALDH bio-sniffer. (**B**) The calibration curve based on the plateau value of fluorescence change at each *trans*-2-nonenal concentration.

Figure 4A shows the response curves of the ALDH bio-sniffer to various concentrations of the *trans*-2-nonenal vapor. A significant increase and stable value in fluorescence output with *trans*-2-nonenal vapor and a recovery to the initial value when the gas supply is stopped were observed. It can be observed that the Δintensity has not completely converged to a steady-state value in the 1.1 ppm and 2.6 ppm of the *trans*-2-nonenal measurements. Therefore, in order to obtain values as close to the convergence value as possible even at these concentrations, we calculated the average Δintensity value at 8–9 min after the start of the sample application and used those values for evaluation of quantitative characteristics (see Figure 4B). The dynamic range of the ALDH bio-sniffer was 0.4–7.5 ppm, which included a concentration range of 2.6 ± 3.5 ppm (mean ± SD) that is reported as the 2-nonenal concentration range emitted from 40–70-year-old subjects [10]. The calibration equation (R^2^ = 0.994) is shown in Equation (4). The theoretical limit of detection (LOD) and quantification (LOQ), calculated from 3 and 10 times the standard deviation values of baseline values, were determined to be 0.23 and 0.26 ppm, respectively.

The ER1 biosensor was also employed to measure *trans*-2-nonanal vapor; however, the signal was only observable at concentrations of 0.4 and 0.8 ppm (refer to Figure S3). This limitation is likely attributed to an issue with the ER1 immobilization method (PMEH entrapment). To achieve quantitative measurements with the highly selective ER1 bio-sniffer, optimization of the immobilization method will be pursued in future studies.

Note that ALDH and ER1 bio-sniffers could be used continuously for at least 3 h. On the other hand, long-term experiments are needed to determine the maximum usable time.

(4) $$avg.\;\;\Delta intensity\;\left(\mathrm{cps}\right)=8449.6\times {\left[\mathrm{trans}-2-\mathrm{nonenal}\;\mathrm{conc}.\;(\mathrm{ppm})\right]}^{0.7624}$$

### 3.4. Signal of Headspace Gases Acquired from Skin-Wiped Cotton at Different Body Sites

**Figure 5:**
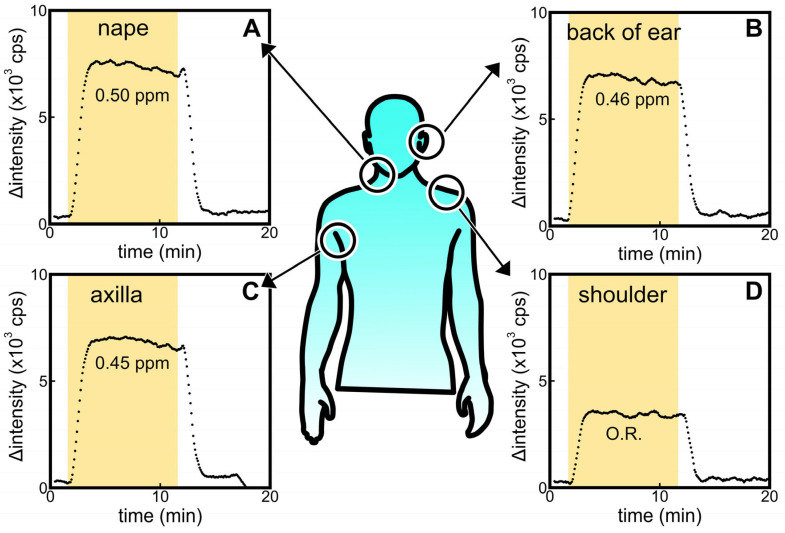
Comparisons of ALDH biosensor signals against headspace gas sample obtained from alcohol-absorbed cotton wiped on the (**A**) nape, (**B**) back of ear, (**C**) axilla, and (**D**) shoulder of participants in their 20s. O.R. means the signal was out of range in the calibration curve.

Figure 5 shows the signals obtained from the ALDH bio-sniffer using samples collected from various sites (A: nape, B: back of the ear, C: axilla, and D: shoulder) of a male participant in their 20s. The participant refrained from bathing and washing his body for 3 days prior to the sample collection. As a result, fluorescent signals were observed at sites other than the shoulder. However, it is important to note that these signals cannot be attributed solely to *trans*-2-nonenal due to the broad catalytic capacity of ALDH for various aldehydes. Considering that hexanal, nonanal, octanal, and decanal are detected with high frequency regardless of age [9,46], the experimental results in Figure 5 may be defined as the baseline for the ALDH biosensor for human samples.

### 3.5. Difference of Signal between Participants in Their 20s and 50s

**Figure 6:**
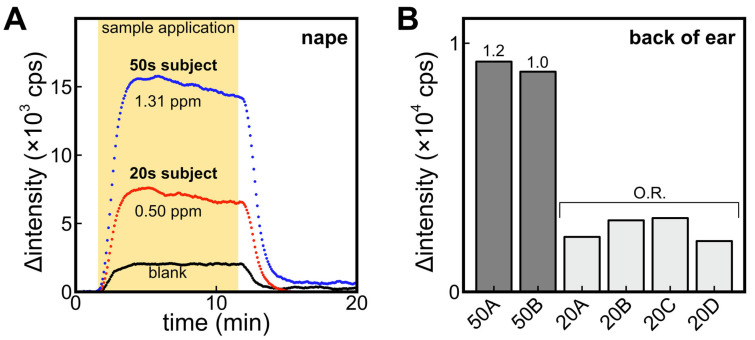
(**A**) Comparison of ALDH biosensor signals against headspace gas sample obtained from nape-wiped alcohol-absorbed cotton of participants in their 20s and 50s. Blank refers to the signal from headspace gas samples of alcohol-absorbed cotton without body wiping. (**B**) Comparison of signals of headspace gas of alcohol-absorbed cotton wiped on the back of ear of participants in their 50s and 20s. O.R. means the signal was out of range in the calibration curve.

In this comparison, participants in their 20s and 50s were instructed to refrain from washing their bodies after bathing the night before the sample collection. Figure 6A shows the signal obtained from the ALDH bio-sniffer using samples collected from the nape of participants in their 20s and 50s. Additionally, a blank sample that is headspace gas from a cotton gauze soaked in 50% ethanol was included for measurement. The results revealed a twofold higher fluorescence output from samples obtained from participants in their 50s. Previous studies have indicated that among aldehydes, the detection frequency of 2-nonenal significantly increases with age [10], and the nape region has been reported to be a site where 2-nonenal is more likely to be generated [26]. Therefore, the age-dependent increase in output observed in this study may be attributed to 2-nonenal. It is important to note that in the previous section, participants in their 20s refrained from body washing for a period of 3 days, and no significant difference was observed compared to the results of this experiment where the prohibition of body washing was limited to one day. Further experiments involving participants in their 50s are deemed necessary to investigate changes in *trans*-2-nonenal concentration due to sebum accumulation.

Figure 6B compares the signal of the ALDH bio-sniffer obtained using headspace gas samples collected from the back of the ear in several participants. Similar age-related differences were observed on the back of the ear, which is characterized by a higher number of sebaceous glands and active sebum secretion, akin to the observations made on the nape region.

### 3.6. Limitations and Advantages of the Developed Sensors

As previously mentioned, the ALDH bio-sniffer exhibited limited selectivity for *trans*-2-nonenal. Therefore, it is crucial to further investigate whether the age-related output difference observed was specifically attributed to *trans*-2-nonenal by considering the outputs from other sensors, such as ER1. Furthermore, the examination of temporal changes was not achieved in this study as headspace gas was utilized. However, with enhancements in sensor sensitivity, the bio-sniffer could be employed for continuous monitoring of skin gas components using a skin gas collection cell, as demonstrated in previous research [47]. This would enable a more comprehensive understanding of the dynamics and variations in skin gas composition over time.

## 4. Conclusions

In this study, our focus was on developing a gas-phase biosensor for the continuous measurement of *trans*-2-nonenal vapor. Initially, we compared two NAD(P)H-dependent enzymes, ALDH and ER1, which exhibited catalytic activity towards *trans*-2-nonenal in both liquid and gas phases. The ALDH biosensor and bio-sniffer demonstrated high sensitivity but low selectivity for *trans*-2-nonenal, while ER1 displayed the opposite characteristics to ALDH, being more selective but less sensitive.

The dynamic range of the ALDH bio-sniffer for *trans*-2-nonenal was determined to be 0.4–7.5 ppm, which encompasses the range of 2-nonenal concentration reported in the previous study [10]. When comparing the outputs of headspace gas samples collected from participants of different ages using the ALDH bio-sniffer, significant differences were observed between samples from participants in their 20s and 50s. It has been reported that in terms of detection rate, 2-nonenal is the only aldehyde component that undergoes significant changes with age [10], suggesting that the observed output difference may be attributed to 2-nonenal. However, a quantitative analysis of each aldehyde concentration was not conducted, and further investigation is required to confirm whether the observed output is specifically derived from 2-nonenal. One potential solution is to utilize the logical product (AND) of the outputs from the ALDH and ER1 bio-sniffers. Statistical analysis methods, including deep learning approaches, could also be employed to effectively combine data from multiple sensors [48].

Bio-sniffer technology provides us with the capability to measure concentration changes over time, and we aim to expand its application to the continuous measurement of various skin components, including *trans*-2-nonenal, in future studies.

## Data Availability

The data that support the findings of this study are available from the corresponding author (K.M.) upon reasonable request.

## Supplementary Materials

The following supporting information can be downloaded at: https://www.mdpi.com/article/10.3390/s23135857/s1, Figure S1: A preparation method of an ALDH-immobilized membrane; Figure S2: Experimental setup for measurement of trans-2-nonenal vapor; Figure S3: Time course of fluorescence intensity by applying trans-2-nonenal vapor against ER1 bio-sniffer.
