# Supplementary material for: Gas-Phase Biosensors (Bio-Sniffers) for Measurement of 2-Nonenal, the Causative Volatile Molecule of Human Aging-Related Body Odor
Source: Sensors (Basel). 2023 Jun 24;23(13):5857. doi: 10.3390/s23135857 (PMC10346471; doi:10.3390/s23135857)
Supplement: Supplementary file 1 [file sensors-23-05857-s001.zip › sensors-2402611-supplementary.pdf]

Supporting Information for

Gas-phase biosensors (bio-sniffers) toward measurement of the causative volatile molecule of human aging odor 2-nonenal

Kenta Iitani[1], Hidehisa Mori[2], Kenta Ichikawa[1], Koji Toma[1,3], Takahiro Arakawa[1,4], Yasuhiko Iwasaki[5], and Kohji Mitsubayashi[1,2,*]

[1] Department of Biomedical Devices and Instrumentation, Institute of Biomaterials and Bioengineering, Tokyo Medical and Dental University, 2-3-10 Kanda-Surugadai, Chiyoda-ku, Tokyo 101-0062, Japan

[2] Graduate School of Medical and Dental Sciences, Tokyo Medical and Dental University, 1-5-45 Yushima, Bunkyo-ku, Tokyo 113-8510, Japan

[2] Department of Electronic Engineering, College of Engineering, Shibaura Institute of Technology, 3-7-5 Toyosu, Koto-ku, Tokyo 135-8548, Japan

[4] Department of Electric and Electronic Engineering, Tokyo University of Technology, 1404-1 Katakura, Hachioji City, Tokyo 192-0982, Japan

[5] Fuculty of Chemistry, Materials and Bioengineering, Kansai University, 3-3-35 Yamate-Cho, Suita-Shi, Osaka 564-0836, Japan

*Corresponding author: Kohji Mitsubayashi

Email: m.bdi@tmd.ac.jp

This PDF file includes:

Figures S1, Figure S2, Figure S3

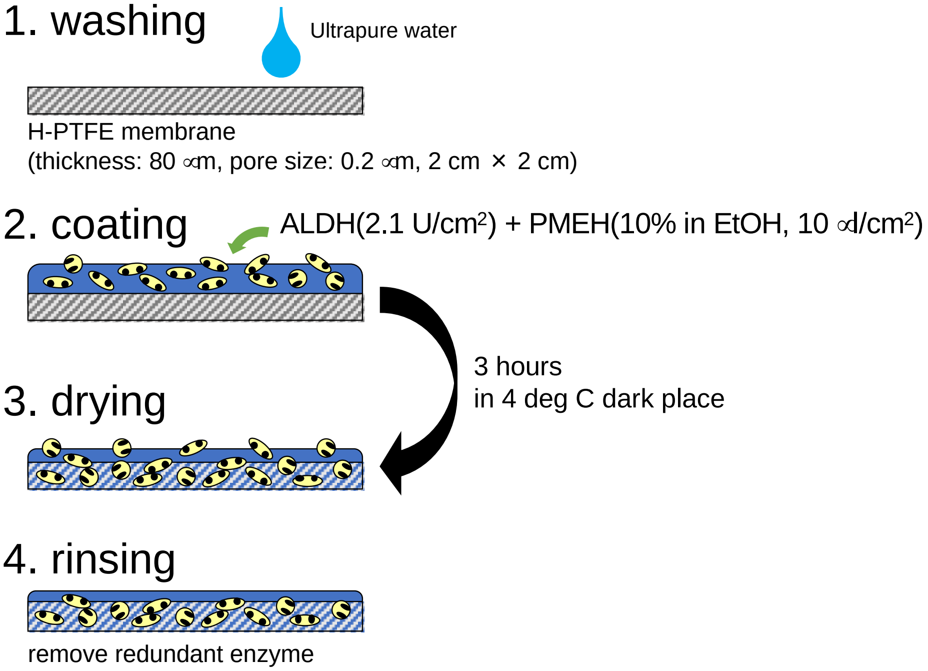

Figure S1. A preparation method of an ALDH-immobilized membrane

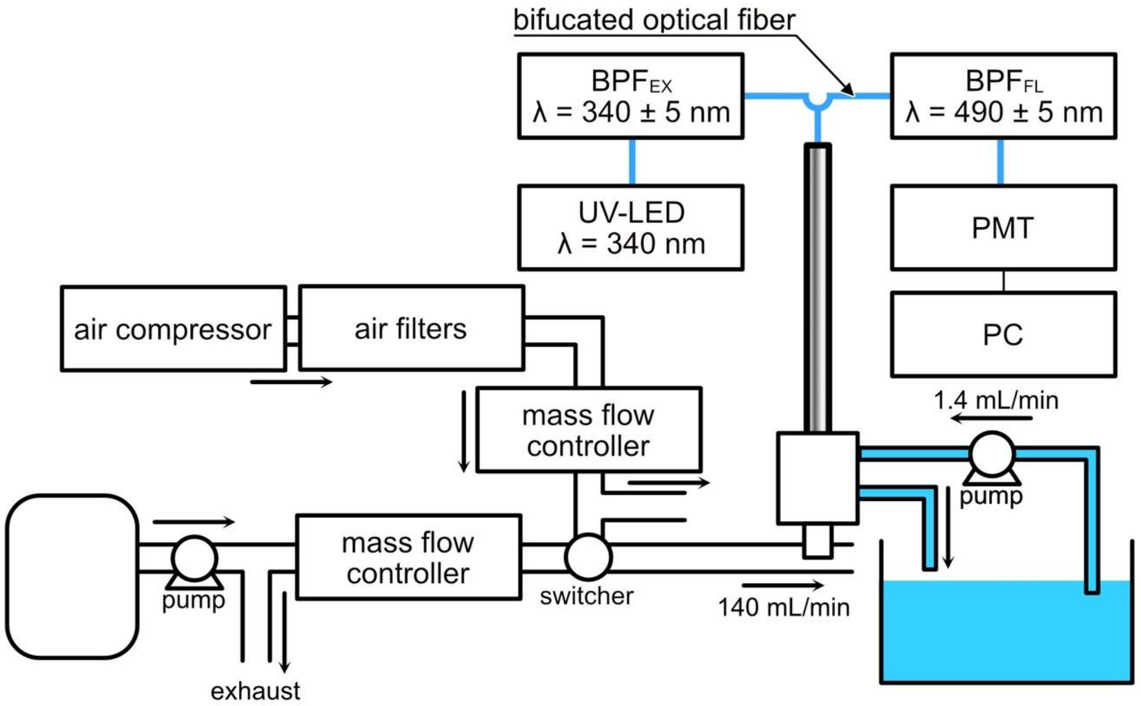

Figure S2. Experimental setup for measurement of *trans*-2-nonenal vapor

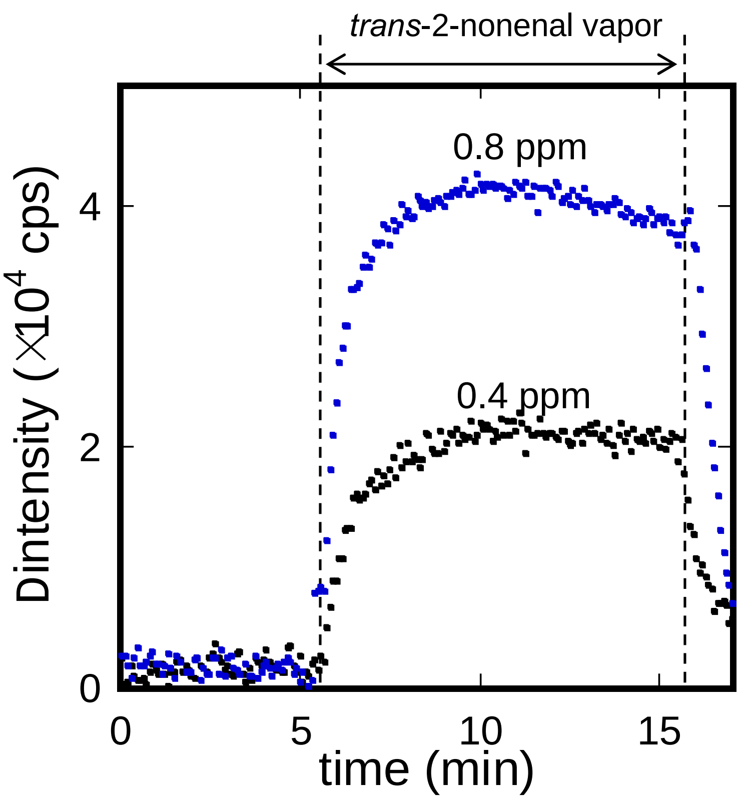

Figure S3. Time course of fluorescence intensity by applying *trans*-2-nonenal vapor against ER1 bio-sniffer.
